# Long-term benefit of pallidal deep brain stimulation in a patient with *VPS16*-associated dystonia

**DOI:** 10.1186/s42466-022-00185-w

**Published:** 2022-05-30

**Authors:** Jan Niklas Petry-Schmelzer, Joohyun Park, Tobias B. Haack, Veerle Visser-Vandewalle, Michael T. Barbe, Gilbert Wunderlich

**Affiliations:** 1 Faculty of Medicine and University Hospital Cologne, Department of Neurology, University of Cologne, Kerpener Staße 62, 50837 Cologne, Germany; 2 Institute of Medical Genetics and Applied Genomics, University of Tuebingen, Tuebingen, Germany; 3 Faculty of Medicine and University Hospital Cologne, Department of Stereotactical and Functional Neurosurgery, University of Cologne, Cologne, Germany; 4 Faculty of Medicine and University Hospital Cologne, Center for Rare Diseases, University of Cologne, Cologne, Germany; 5 Centre for Rare Diseases, University of Tuebingen, Tuebingen, Germany

**Keywords:** VPS16, Deep brain stimulation

Here, we report a patient with segmental dystonia harboring a heterozygous pathogenic variant in *VPS16* who underwent pallidal deep brain stimulation with sustained tremendous benefit over a follow-up period of 3 years.

## Case report

A 43-year-old patient presented with segmental dystonia beginning with mild writer’s cramp of the right hand at the age of 33 years. At the age of 40 years, he first recognized a torticollis to the left. The severity of cervical dystonia progressed over the years, while writer’s cramp remained mild but disabling. Further medical and family history was unremarkable. The patient has two healthy children.

On physical examination we observed a retrocaput and torticollis to the left of variable severity. There was no limitation in head movements. Writing with the right hand induced a mild writer’s cramp. The patient was able to suppress cervical dystonia by a “geste antagoniste”, specifically by light touch of his chin with his fingers. Additionally, the patient reported light improvement of dystonic symptoms by alcohol consumption. (Burke-Fahn-Marsden Dystonia Rating Scale—Movement Scale (BFMDRS): 16 points, Toronto Western Spasmodic Torticollis Rating Scale—Torticollis Severity Scale (TWSTRS-I): 16 points,—Disability Scale (TWSTRS-II): 13 points,—Pain Scale (TWSTRS-III): 5 points). Furthermore, the patient suffered of mild to moderate anxiety and depression. As per clinical routine, the patient underwent psychiatric evaluation prior to deep brain stimulation, determining these non-motor symptoms as reactive to his dystonic symptom burden. Neuropsychiatric examination showed mild mnestic (PANDA 23/30 points) but no cognitive impairment. Laboratory examination was not suspicious for any secondary cause of dystonia. Exome sequencing identified a heterozygous predicted loss of function variant (pLoF) in the canonical sequence of *VPS16* ((ENST00000380445.3 (NM_022575.3)): c.1903C > T, p.Arg635Ter). This variant is listed once in the gnomAD browsers (gnomad.broadinstitute.org) in a heterozygous state (age not given). However, it has also been identified in an independent, similarly affected patient, and is predicted to be deleterious by the CADD pathogenicity predictor (CADD score 37, https://cadd.gs.washington.edu/) [1]. Other heterozygous pLoFs have been reported to cause dystonia as well, and case–control studies have shown significant enrichment of pLoFs in dystonia patients [1, 2]. Thus, we classified the variant as pathogenic according to the recommendations of the American College of Medical Genetics and Genomics and the Association for Molecular Pathology (ACMG class 5; PVS1, PS1, PS4) [3]. Family members of our patient were not available for additional segregation analysis.

**Fig. 1:**
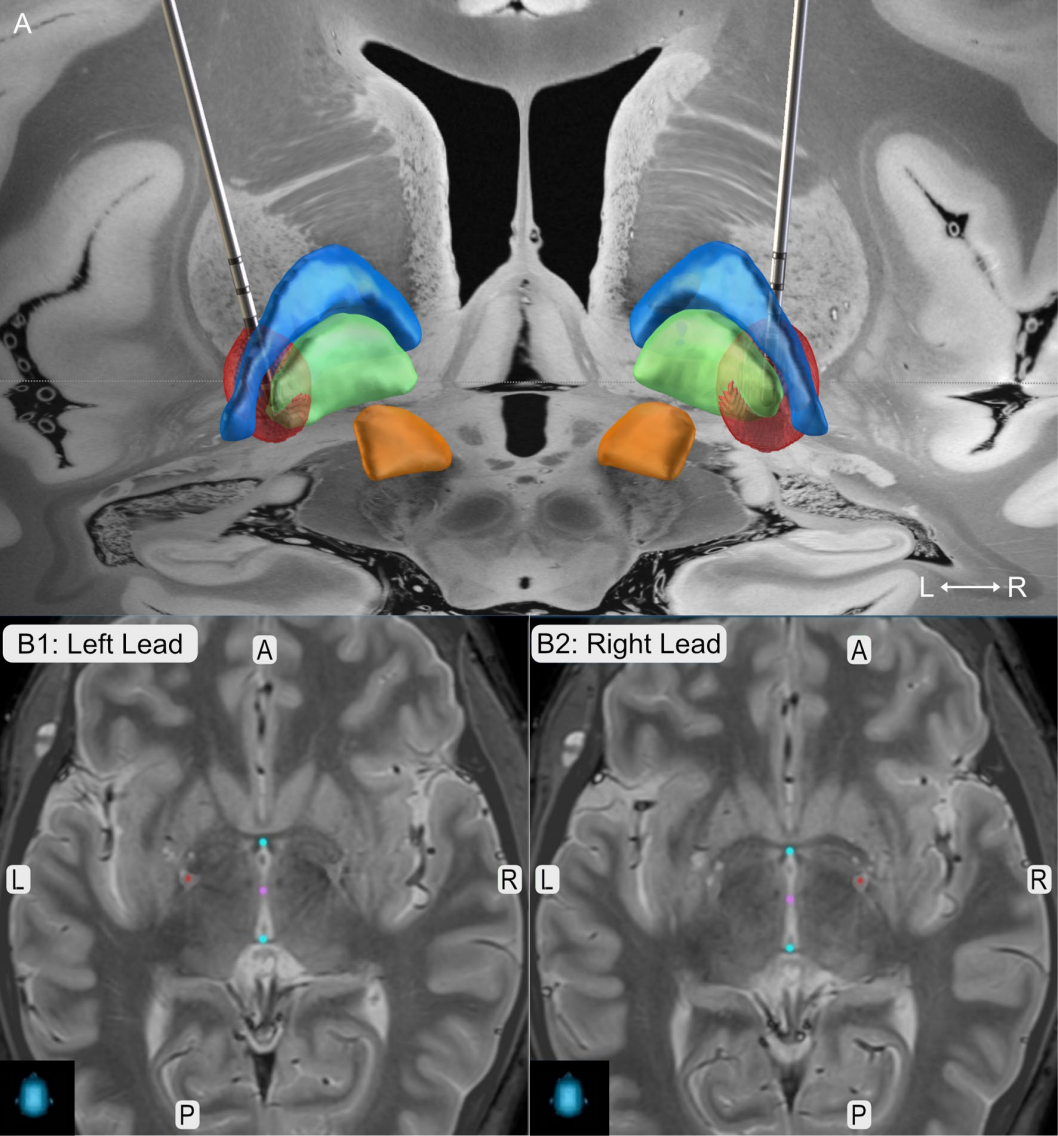
Lead Position. **a** Lead reconstruction in MNI ICBM 2009b space as implemented in LEAD DBS. Leads are shown in posterior view together with the globus pallidus externus (blue), globus pallidus internus (green) and the subthalamic nucleus (orange) as included in the DISTAL atlas [10, 11]. Red balls illustrate the local stimulation spread of 3-year Follow-Up stimulation parameters (Left: C+, 1-(50%), 2-(18%), 3-(16%), 4-(16%), 60 µs, 104 Hz, 4.2 mA; Right: C+, 1-(50%), 2-(18%), 3-(16%), 4-(16%), 60 µs, 104 Hz, 5.7 mA, Boston Scientific Vercise Directed lead). (B) Lead positions (red dots) as extracted fromStealthViz™, Medtronic in axial view. Coordinates in relation to AC-PC (blue dots) are x = -21.6 mm, y = -1.7 mm, z = -17.6 mm for the left lead, and x = 17.7 mm, y = 3.0 mm, z = 11.7 mm for the right lead. Abbreviations: A = anterior, L = left, P = posterior, R = right

The patient only poorly responded to trihexyphenidyl and botulinum toxin and therefore underwent pallidal deep brain stimulation (DBS) after exclusion of any contraindications and evaluation of the case in our interdisciplinary expert board. Dystonic symptoms including writer’s cramp and non-motor symptoms improved tremendously under DBS with a sustained response over three years without any additional medication needed (see Additional file 1, 3-year follow-up: BFMDRS: 1 point TWSTRS-I: 0 points, TWSTRS-II: 1 point, TWSTRS-III: 4 points). There were no stimulation-induced side-effects reported. The lead position is illustrated in Fig. 1.

## Discussion

*VPS16* encodes vacuolar protein sorting associated protein 16, a key component of the two tethering protein complexes CORVET (class C core vacuole/endosome tethering) and HOPS (homotypic fusion and vacuole protein sorting). The link between malfunction of these protein complexes causing defective endosomal maturation and/or lysosomal dysfunctions and inherited dystonia has also been described in *VPS41* and *VPS11* mutations, leading to the subsummation as HOPS-associated neurological disorders (HOPSANDs) [2, 4]. In 2016, Cai et al. were the first to report a homozygous missense variant (c.156C > A, p.Asn52Lys) in *VPS16* in a consanguineous family with adolescent-onset dystonia and five affected family members. In addition, they were able to reproduce the observed phenotype-genotype correlation in a mouse-model [5]. In 2020, Steel et al. reported 18 cases with heterozygous pLoFs and one case with a microdeletion spanning *VPS16* [2]. Recently, Park et al. were able to demonstrate that pLoFs in *VPS16* associated with dystonia were in the same highly expressed canonical transcript (ENST00000380445.3 (NM_022575.3)) [1]. Regarding the pattern of inheritance, a dominant inheritance with incomplete penetrance but also de novo occurrence has been reported [2, 6].

Overall, 26 affected individuals with heterozygous pLoFs in VPS16 have been reported so far [2, 5–8]. The prevalence in early onset dystonia has been estimated to 0.9 to 4% [2, 6]. Most of the patients presenting with segmental dystonia comprised writer’s cramp and cervical, oromandibular or limb dystonia with a median age at onset of 12 years (range: 3–50 years), and progression over the years. Additional features reported are mild to moderate intellectual disability (~ 20%), neuropsychiatric symptoms (~ 30%) seizures (< 10%), and only in single cases other movement disorders such as myoclonic jerks [1, 2, 6]. In some patients, symptom relief was observed with Levodopa (4/26), whereas trihexyphenidyl was effective in only one patient. Botulinum toxin was successfully used in 8/26 patients, especially to treat cervical dystonia. Including the present case, 7 patients were reported to receive pallidal DBS so far. While four of them showed significant symptom improvement one patient did only partly benefit and two patients did not benefit from the intervention. Besides the sustained treatment effect over 3 years, as reported here, only one other patient has been reported regarding longtime follow-up with a sustained treatment effect over 7 years [2, 6]. While no distinct pattern of dystonic symptoms of patients not responding to DBS could be identified from the literature, DBS non-responder seem to have a younger age at onset (3, 7 and 10 years), than patients responding well to DBS (age at onset: 11, 16, 19, 33 years). This dependency of treatment response on age at onset has also been reported for other monogenic dystonias, in terms of worse motor outcomes with older age at onset in DYT-TOR1A and younger age at onset in DYT-SGCE [9].

To conclude, this case report demonstrates sustained response to pallidal DBS in rare *VPS16-*associated dystonia, and adds to individualized counseling of patients with dystonia prior to DBS surgery.

## Supplementary Information

**Additional file 1: Video 1**. Response to pallidal deep brain stimulation. Preoperatively the patient presented with cervical dystonia and writer’s cramp on the right. After pallidal DBS the patient improved tremendously with sustained benefit over three years.

## Abbreviations

DBS: Deep brain stimulation
TWSTRS: Torticollis Rating Scale
BFMDRS: Burke-Fahn-Marsden Dstonia Rating Scale
